# Increases in the susceptibility of human endometrial CD4^+^ T cells to HIV-1 infection post-menopause are not dependent on greater viral receptor expression frequency

**DOI:** 10.3389/fimmu.2024.1506653

**Published:** 2025-01-13

**Authors:** Landon G. vom Steeg, Zheng Shen, Jane Collins, Mickey V. Patel, Fiona D. Barr, Daniel C. Hopkins, Christina Ochsenbauer, Charles R. Wira

**Affiliations:** ^1^ Department of Microbiology and Immunology, Geisel School of Medicine at Dartmouth, Lebanon, NH, United States; ^2^ Department of Medicine, Heersink School of Medicine, University of Alabama at Birmingham, Birmingham, AL, United States

**Keywords:** HIV-1, menopause, CD4^+^ T cells, macrophage, human female reproductive tract

## Abstract

Epidemiological evidence suggests that post-menopausal women are more susceptible to HIV infection following sexual intercourse than are younger cohorts for reasons that remain unclear. Here, we evaluated how menopause-associated changes in CD4^+^ T cell numbers and subsets as well as HIV coreceptor expression, particularly CCR5, in the endometrium (EM), endocervix (CX), and ectocervix (ECX) may alter HIV infection susceptibility. Using a tissue-specific mixed cell infection model, we demonstrate that while no changes in CD14^+^ macrophage infection susceptibility were observed, CD4^+^ T cell HIV-1 infection frequency increases following menopause in the EM, but not CX nor ECX. Unexpectedly, the CD4^+^ T cell expression of two known correlates of HIV infection susceptibly, CCR5 and integrin-α4β7, increased following menopause across all three tissues despite only being associated with increased infection frequency in EM derived CD4^+^ T cells. After controlling for changes in the expression of either receptor, both CCR5 and α4β7 expressing CD4^+^ T cells isolated from the EM of post-menopausal women remained more susceptible to HIV-1 infection than those isolated from pre-menopausal women. Shifts in T helper subset composition, including increases in Th1 frequency and decreases in Th17 and Treg frequency were also observed in the EM only following menopause, but did not correlate with increased infection frequency. Treatment of EM derived CD4^+^ T cells with 17β-estradiol (E_2_) prior to viral infection, reduced infection frequency independent of changes in either CCR5 or α4β7 expression frequency. Our results demonstrate that the susceptibility of EM derived CD4^+^ T cells to HIV-1 infection increases post menopause but is unlikely to be driven by increased expression frequency of either CCR5 or integrin-α4β7. These findings contribute to our understanding of how advanced age alters HIV infection risk which will become increasingly important as the human population continues to age.

## Introduction

Globally, women account for nearly half of all new Human Immunodeficiency Virus (HIV) infections (1). Despite general declines in younger age groups (2, 3), the incidence of HIV infection has risen in recent years within older cohorts (4, 5), with those over the age of 45 now accounting for roughly 35% of all new HIV infections among women in the USA (6). HIV infection among older female populations primarily occurs through sexual transmission (7), with epidemiological studies indicating that women over the age of 45 are 3.9-fold and 8.7-fold more likely than younger women to be infected with HIV-1 and HIV-2, respectively, following vaginal intercourse (8, 9). However, the degree to which these observed increases in HIV infection risk are driven by behavioral changes [e.g., reduced condom used and lower perceived infection risk (7, 10, 11)], or biological changes associated with increasing age, remains unclear.

CD4^+^ T cells and macrophages are the primary targets of HIV infection in the mucosa of the female reproductive tract (FRT) (12–14), with transmitted/founder virus envelope-proteins generally requiring high levels of CD4 together with the CCR5 coreceptor, thus suggestive of preferential CD4^+^ T cell infection (12). In addition to CCR5, differential expression of alternate coreceptors, including integrin α4β7 (15, 16), CX3CR1 (17), and CXCR4 (18), have been suggested to play intricate roles in the establishment of productive infection either indirectly or through direct interactions with viral envelope glycoprotein 120. Likewise, changes in CD4^+^ T cell activation state has also been reported to modulate HIV entry and infection susceptibility with CD4^+^ T cells expressing the early activation marker CD69 being preferential targets of HIV infection relative to naïve cells in both blood and tissue (19–23). Moreover, heterogeneity in functional T helper cell phenotype similarly influences HIV entry and infection susceptibility with CCR6 expressing CD4^+^ T cells, notably Th17 and potentially Th22 cells, being preferential targets of HIV infection in the bloodstream and mucosa of the FRT (24–27). Given the above effects of immune heterogeneity on cellular susceptibility to HIV infection, understanding how life stage events, including menopause and the years following menopause, alter the immune system and thus HIV infection risk are critically important.

Menopause typically occurs during the 4^th^ and 5^th^ decades of life and is associated with profound hormonal declines and immune changes in the FRT (28–31). In addition to reductions in epithelial cell barrier function and the production of innate antiviral mediators (30, 32, 33), direct changes in markers of CD4^+^ T cell HIV susceptibility have also been reported following menopause. Notably, prior research suggests the expression of CCR5 is elevated on CD4^+^ T cells isolated from both PBMCs and the tissues of the FRT with advanced age (25, 34, 35), with greater CD4^+^ T cell CCR5 expression hypothesized to promote increased HIV infection susceptibility post-menopause. Likewise, we and others have previously found the frequency of CCR6^+^ CD4^+^ T cells to be greater in the EM of post-menopausal women (25) and in the bloodstream of individuals over the age of 50 (36). While these observed menopause-associated changes in immune function and phenotype suggest biological factors may play a significant role in increases in HIV infection susceptibility, few studies have directly evaluated how menopause alters the HIV-infection susceptibility of macrophages and CD4^+^ T cells in the FRT.

Herein, we use a human mixed cell suspension model to directly assess whether the susceptibility of FRT CD4^+^ T cells and CD14^+^ macrophages to HIV-1 infection is altered in the years following menopause and test the hypothesis that greater CD4^+^ T cell CCR5 expression following menopause results in increased HIV-1 infection susceptibility in the FRT. We demonstrate that CD4^+^ T cell susceptibility to HIV infection is enhanced in the EM, but not the CX and ECX, following menopause. This was associated with, but is not likely explained by, increases in previously identified determinants of CD4^+^ T cell HIV infection susceptibility including CCR5 and integrin α4β7 expression. These findings suggest that aging associated increases in female HIV infection susceptibility are, at least in part, driven by biological changes that occur in the years following menopause.

## Materials and methods

### Study participants

All investigations involving human subjects were approved by the Dartmouth College Committee for the Protection of Human Subjects, Dartmouth-Hitchcock Medical Center (DHMC), and the Dartmouth Health Institutional Review Board. Informed written consent was obtained from all patients prior to surgery and all research was conducted in accordance with the policies of the Declaration of Helsinki. Endometrial (EM), endocervical (CX), and ectocervical (ECX) tissues were obtained from patients undergoing surgical hysterectomy at DHMC (Lebanon, NH, USA) for benign indications. Patients with evidence of reproductive tract infection, reproductive tract cancer, who were taking hormonal birth control, or had a recent history of exogenous hormone therapy were excluded from this study. All tissue received was distal to any sites of pathology. Menopausal status and menstrual cycle stage were determined by trained clinical pathologists via histological assessment with post-menopausal status defined as patients with atrophic EM. Pre-menopausal status was assigned as those patients with histological evidence of normal menstrual cycling. The mean age of pre-menopausal women was 37.8 years (SD = 8.6; range: 22-51) and the mean age of post-menopausal women was 66.5 years (SD = 7.7; range: 52-80).

### Tissue processing

After surgery, fresh tissues were promptly processed, sectioned, and assessed for inclusion criteria by the staff of the DHMC Department of Pathology and Laboratory Medicine prior to transfer to our laboratory. Mucosal tissue sections were then weighed with mean tissue mass received measured as 3.0 g (95% CI: 2.3 to 3.7 g) for pre-menopausal EM tissue, 1.8 g (95% CI: 1.4 to 2.1 g) for post-menopausal EM tissue, 1.6 g (95% CI: 1.2 to 2.0 g) for pre-menopausal CX tissue, 1.4 g (95% CI: 1.1 to 1.7 g) for post-menopausal CX tissue, 2.5 g (95% CI: 1.8 to 3.3 g) for pre-menopausal ECX tissue and 1.4 g (95% CI: 1.0 to 1.7 g) for post-menopausal ECX tissue.

As described previously (25, 37), samples were then minced under aseptic conditions into 1-2 mm fragments using sterile scalpels (Feather, Osaka, Japan) before being enzymatically digested in a HBSS solution containing 0.05% Type 1V collagenase (Sigma-Aldrich, St, Louis, MO, USA) and 0.01% DNase (Worthington Biochemical, Lakewood, MO, USA) 37°C. After 1 h incubation, digested tissues were passed through 200 μm followed by 35 μm nylon mesh filters (LBA, Miami, FL, USA) to remove debris and epithelial cells sheets. Red blood cells were then removed via osmotic lyses, dead cells were removed using a commercial dead cell removal kit (Miltenyi Biotec, Auburn, CA, USA), after which the flow through was again filtered using a 30 μm nylon filter (Miltenyi Biotec). The resulting mixed cell suspensions contained immune cells, stromal fibroblasts, and residual single epithelial cells. Total numbers of live cells were determined by hemacytometer and trypan blue (VWR-Amresco, Solon, OH, USA) exclusion.

### Virus and HIV-1-infection

A previously described pNL-GFPm.6ATRi-BaL.ecto reporter virus (38) was used for all infections. This CCR5 trophic virus, referred to as “BaL-GFP” throughout this paper, was produced through 293T cell transfection and only expresses GFP in productively infected HIV-1-susceptible cells. The TZM-bl cell line was used to determine the infectious titer of all virus stocks prior to experimental use as previously described (39).

Unstimulated mixed cell solutions were resuspended in X-VIVO 15 media (Lonza, Walkersville, MD, USA) supplemented with 10% charcoal dextran stripped human serum (Valley Biomedical, Winchester, VA, USA) and plated out in triplicate for each sample at a density of 300,000 cells/well in a round bottom ultra-low attachment 96-well plate (Corning, Corning, NY, USA). In one well for each tissue sample, 10 μM of the reverse transcriptase inhibitor Azidothymidine (AZT; NIH HIV Reagent Program) was added as a viral infection control and cells were incubated for 2 h at 37°C. Cells were then infected at an MOI of 1 (based on TZM-bl titer) with HIV-1 BaL-GFP with uninfected control wells receiving medium without virus. After 2 h of virus exposure, all cells were washed twice with media to remove residual virus and cell cultures kept at 37°C for 6 days with AZT at a concentration of 10 μM added to replication control wells. On days 3 and 5 post infection, half the media was removed from each well and replaced with fresh media and with AZT at a concentration of 10 μM again added to replication control wells.

### Flow cytometry

After 6 days in culture, mixed cell suspensions were stained with combinations of the following anti-human antibodies: CXCR4-PE (Clone 12G5; BD), CX3CR1-PE/Dazzle (Clone 2A9-1; BioLegend), CCR5-PerCPCy5.5 (Clone J418F1; BioLegend), α4β7-AF647 (R&D Systems), CD45-AF700 (Clone 2D1; BioLegend), CD3-APC/E780 (Clone UCHT1; BioLegend), CD8-VF450 (Clone SK1; Tonbo Biosciences), CD69-VioGreen (Clone REA824, Miltenyi Biotec), CCR10-PE (Clone 6588-5; BioLegend), CCR6-PE/Dazzle (Clone G034E3; BioLegend), CXCR3-PE/Cy5 (Clone G025H7; BioLegend), CCR4-PE/Cy7 (Clone L291H4; BioLegend), CD127-APC (Clone A019D5; BioLegend), CD25-APC/Cy7 (Clone BC96; BioLegend), CD3-E450 (Clone SK7; Invitrogen), CD8-BV510 (Clone SK1; BioLegend), CD45-PC5.5 (Clone J33; IOTest), and CD14-PE/Vio770 (Clone TÜK4; Miltenyi Biotec). Following staining, cells were fixed with 2% methanol free formaldehyde (Polysciences Inc., Warrington, PA, USA) and data acquired using a 10-color Gallios Flow Cytometer (Beckman Coulter Life Sciences, Indianapolis, IN, USA) running Kaluza software. Data analysis was performed using FlowJo v10.10 Software (Tree Star, Inc., Ashland, OR, USA).

### Estrogen treatment

For select experiments tissue-specific mixed cell suspensions were generated from pre- and post-menopausal patients, with a portion of post-menopausal cells incubated for 2 h with 17β-estradiol (E_2_; Sigma-Aldrich, Burlington, MA, USA) at a concentration of 5 x 10^-8^ M prior to HIV-1 BaL-GFP infection. E_2_ exposure at a concentration of 5 x 10^-8^ M was maintained throughout the 6-day post-infection period with E_2_ prepared as described previously (40).

### Data analysis

**Figure 1:**
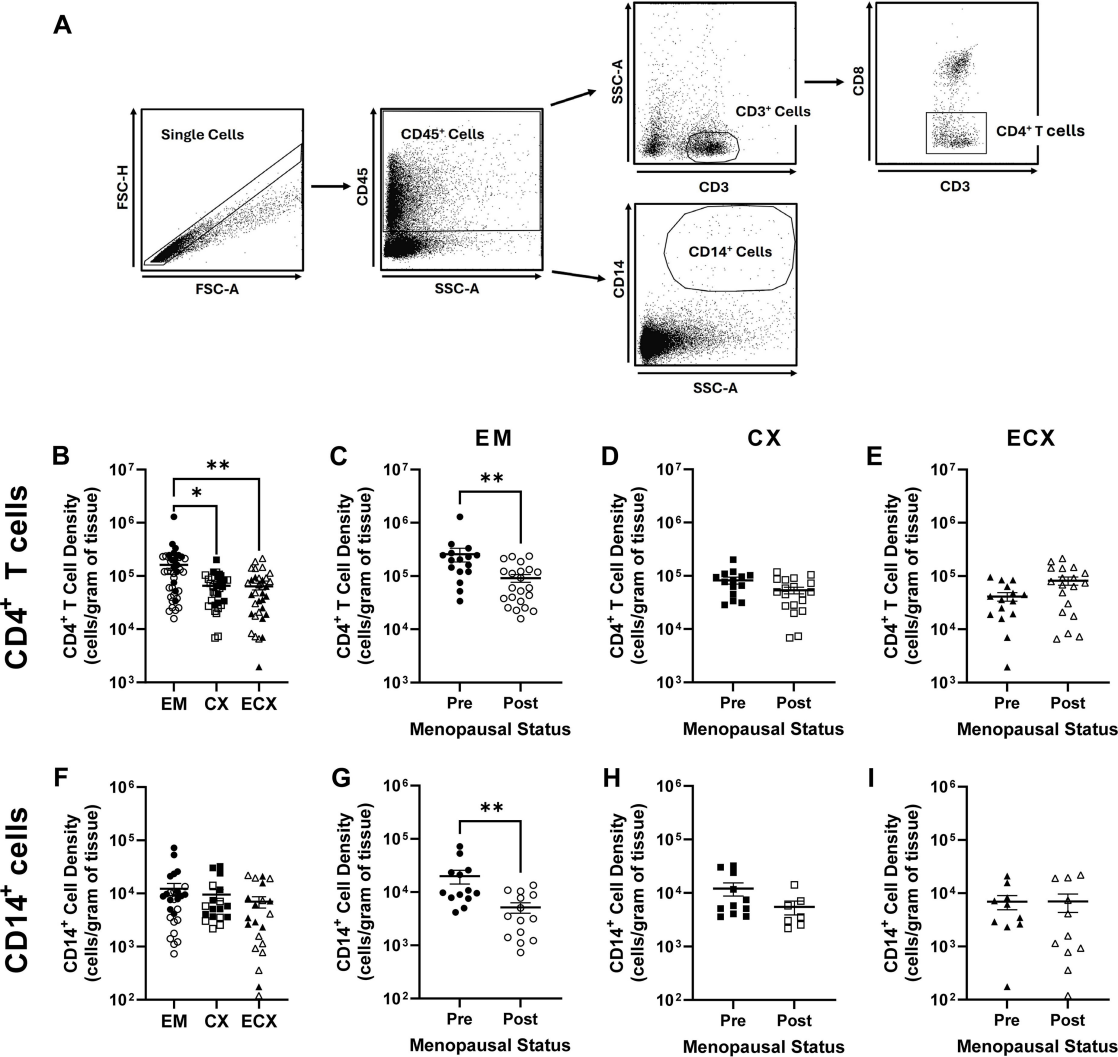
Effect of menopausal status on mucosal HIV-1-infection target cell density in the EM, CX, and ECX. Gating strategy for the identification of CD4^+^ T cells and CD14^+^ cells **(A)**. Relative mucosal CD4^+^ T cell density in the EM, CX, and ECX **(B)**. Effect of menopausal status on mucosal CD4^+^ T cell density in the EM **(C)**, CX **(D)**, and ECX **(E)**. Relative mucosal CD14^+^ cell density in the EM, CX, and ECX **(F)**. Effect of menopausal status on CD14^+^ cell density in the EM **(G)**, CX **(H)**, and ECX **(I)**. Individual patient data are shown with circles for EM tissue, with squares for CX tissue, and with triangles for ECX tissue. Solid shapes indicate samples from pre-menopausal patients while open shapes indicate samples from post-menopausal patients. Data represent the mean ± SEM with significant differences determined by Kruskal-Wallis test followed by Dunn’s post-test for pairwise multiple comparisons for **(B, F)**, and Mann-Whitney *U* test for **(C–E, G–I)**. *P < 0.05 and **P < 0.01.

In this study, CD4^+^ T cells were defined as CD45^+^ CD3^+^ CD8^-^ single cells; CD14^+^ macrophages were defined as CD45^+^ CD14^+^ single cells exhibiting high side-scatter (**Figure 1A**). Reported infection frequencies represent the % GFP^+^ cells from HIV-1 infected samples minus the % GFP^+^ cells from the corresponding HIV-1 + AZT treated control to ensure that reported GFP expression is solely the result of productive HIV infection.

Discrete measures were analyzed by Mann-Whitney *U* test, Kruskal-Wallis test followed by Dunn’s post-test for pairwise multiple comparisons, Mann-Whitney *U* test with Holm-Šídák correction for multiple comparisons or 2-way ANOVA with Tukey’s post-test for multiple comparisons. Statistical analyses were performed using GraphPad Prism 10.2.3 software and mean differences were considered significant at two-sided P < 0.05.

## Results

### HIV-infection target cell density decreases in the EM following menopause

The quantity of HIV target cells at the site of mucosal exposure has been proposed to modify HIV infection susceptibility (41). To determine whether menopause alters the density of HIV-1 target cells in the FRT, tissue specific single cell suspensions were generated from mucosal tissues of the EM, CX, and ECX as described in the methods. CD4^+^ T cell and CD14^+^ cell frequencies were subsequently determined by flow cytometry (**Figure 1A**) and used to calculate cell density normalized to cell number per gram of tissue.

Among the three tissue types, mean CD4^+^ T cell density was significantly greater in the EM (1.62 x 10^5^ cells/g) relative to CX (0.65 x 10^5^ cells/g) and ECX (0.64 x 10^5^ cells/g) with density not differing between the latter two tissues (**Figure 1B**). Following stratification by menopausal status, the density of EM (2.58 x 10^5^ cells/g vs 0.92 x 10^5^ cells/g; **Figure 1C**) CD4^+^ T cells was significantly lower in post-menopausal relative to pre-menopausal women. In contrast, menopausal status did not alter CD4^+^ T cell density in the CX (**Figure 1D**) or ECX (**Figure 1E**).

Unlike CD4^+^ T cells, mean CD14^+^ cell density did not significantly differ between the EM (1.23 x 10^4^ cells/g), CX (0.95 x 10^4^ cells/g), or ECX (0.70 x 10^4^ cells/g; **Figure 1F**), though CD14^+^ cell density was significantly lower than CD4^+^ T cell density for all three tissues (**Figure 1A** vs. **Figure 1E**; approximately 10-fold; P > 0.0001). Following stratification by menopausal status, a significant decrease in CD14^+^ cell density was observed in post-menopausal versus pre-menopausal women in the EM only (1.99 x 10^4^ cells/g vs 0.51 x 10^4^ cells/g; **Figure 1G**). In contrast, no differences in CD14^+^ cell density were observed with menopausal status in the CX (**Figure 1H**) or ECX (**Figure 1I**). Taken together these data indicate that the density of HIV-1 target cells decreases in the mucosa of the EM, but not the CX or ECX, following menopause. Moreover, these data suggest that CD4^+^ T cells are the most abundant putative HIV-1 target cell type throughout the mucosa of the FRT.

### CD4^+^ T cell HIV-1-infection frequency increases in the EM following menopause

**Figure 2:**
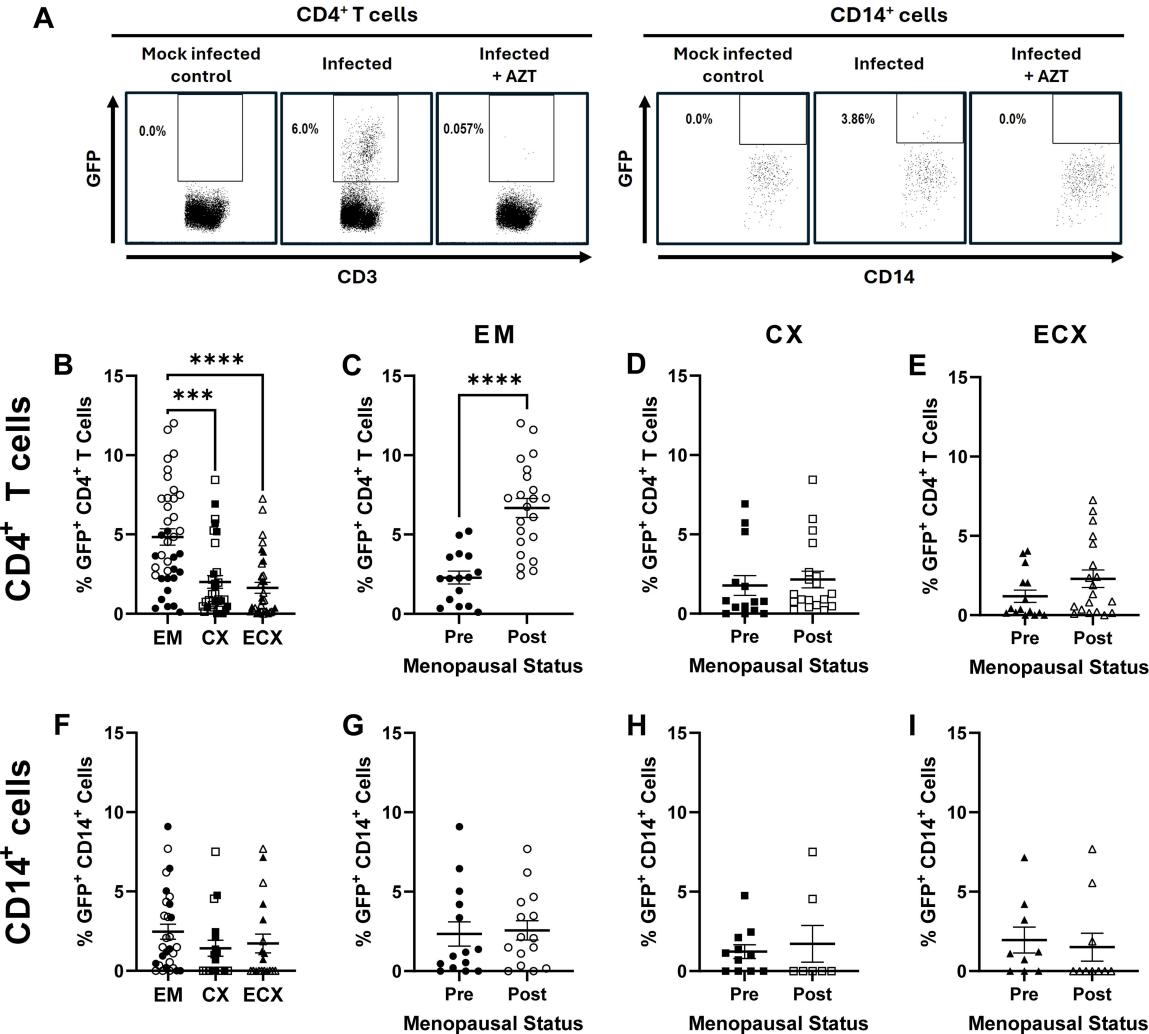
Effect of menopausal status on HIV-1-infection frequency in the EM, CX, and ECX. Female reproductive tract CD4^+^ T cells and CD14^+^ cells express GFP following productive HIV-1 BaL-GFP infection, which is inhibited in the presence of the reverse transcriptase inhibitor AZT **(A)**. Relative mucosal CD4^+^ T cell infection frequency in the EM, CX, and ECX **(B)**. Effect of menopausal status on mucosal CD4^+^ T cell infection frequency in the EM **(C)**, CX **(D)**, and ECX **(E)**. Relative mucosal CD14^+^ cell infection frequency in the EM, CX, and ECX **(F)**. Lack of an effect of menopausal status on CD14^+^ cell infection frequency in the EM **(G)**, CX **(H)**, and ECX **(I)**. Individual patient data are shown with circles for EM tissue, with squares for CX tissue, and with triangles for ECX tissue. Solid shapes indicate samples from pre-menopausal patients while open shapes indicate samples from for post-menopausal patients. Bars represent the mean ± SEM with significant differences determined by Kruskal-Wallis test followed by Dunn’s post-test for pairwise multiple comparisons for **(B, F)**, and Mann-Whitney *U* test for **(C–E, G–I)**. ***P < 0.001, and ****P < 0.0001.

We next assessed whether menopausal status alters the susceptibility of CD4^+^ T cells and CD14^+^ cells to HIV-1 infection in the FRT mucosa. As before, tissue specific mixed cell suspensions were generated from the EM, CX, and ECX prior to either mock infection or infection at a MOI of 1 with a HIV-1 BaL reporter virus that expresses GFP following productive viral infection. After 6 days of viral exposure, flow cytometry was used to determine CD4^+^ T cell and CD14^+^ cell infection frequency in the mixed cell cultures. As seen in (**Figure 2A**), while no GFP expression was detected in mock infected controls, GFP expression in virally infected cells could be readily detected by flow cytometry. Moreover, GFP expression was essentially absent in cells exposed to HIV-1 BaL-GFP in the presence of the reverse transcriptase inhibitor AZT, thus, demonstrating the specificity of detected GFP expression as a marker for *de novo* infection of both CD14^+^ cells and CD4^+^ T cells.

Following six days of viral exposure, mean CD4^+^ T cell HIV-1 infection frequency was significantly greater in the EM (4.8%) relative to the CX (2.0%) or ECX (1.6%; **Figure 2B**), with no differences observed between the CX and ECX. When stratified by menopausal status, mean CD4^+^ T cell infection frequency was significantly greater in the EM of post-menopausal women (6.7%) relative to pre-menopausal women (2.3%; **Figure 2C**), with pre-menopausal EM infection frequencies consistent with those observed for the CX and ECX for both pre- and post-menopausal donors (**Figure 2B**). Thus, despite the changes observed in the EM, menopause did not alter the HIV-1 infection susceptibility of CD4^+^ T cells isolated from the CX (**Figure 2D**) nor ECX (**Figure 2E**) as measured by relative and absolute infection frequency.

In contrast to CD4^+^ T cells, no differences in mean CD14^+^ cell infection frequency were observed between the three tissue types (**Figure 2F**). Likewise, menopausal status did not alter mean CD14^+^ cell infection frequency in the EM (**Figure 2G**), CX (**Figure 2H**) nor ECX (**Figure 2I**). These data suggest that greater CD4^+^ T cell infection frequency following menopause is driven by tissue specific changes unique to the EM.

### Known determinants of CD4^+^ T cell HIV-1 infection susceptibility increase following menopause in the EM, CX, and ECX, and are associated with increased infection frequency in the EM, but not in the CX and ECX

**Figure 3:**
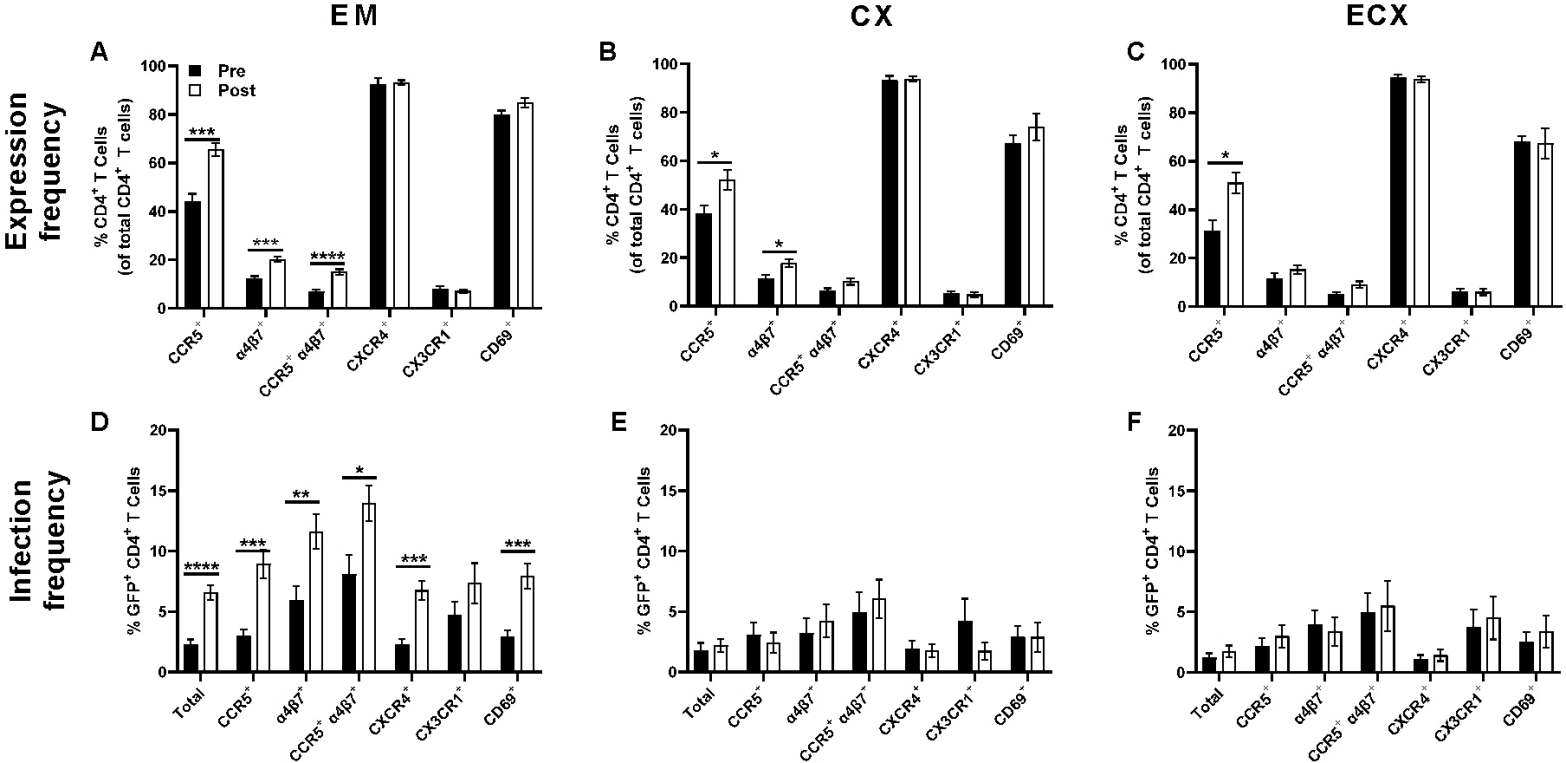
Effect of menopause on the expression of surface markers of CD4^+^ T cell HIV-1 infection susceptibility in the mucosa of the FRT. Menopausal associated changes in the expression of select correlates of HIV-1 infection susceptibility by CD4^+^ T cells in the EM **(A)**, the CX **(B)**, and the ECX **(C)**. The effect of menopause on HIV-1 infection frequency in CD4^+^ T cells expressing select correlates of HIV-1 infection susceptibility in the EM **(D)**, CX **(E)**, and ECX **(F)**. Data from pre-menopausal patients are shown with solid black bars while data from post-menopausal patients are shown with open black bars. Data represent the mean ± SEM with significant differences determined by Mann-Whitney *U* test using the Holm-Šídák correction for multiple comparisons. *P < 0.05, **P < 0.01, ***P < 0.01, and ****P < 0.0001.

Previous work by our group and others suggest that greater CCR5 expression may increase CD4^+^ T cell susceptibility to HIV infection post-menopause (25, 35). To test this hypothesis, we first measured the expression of CCR5 and other select surface markers of CD4^+^ T cell HIV-1 infection susceptibility on cells isolated from the FRT. These included the expression of the alternate coreceptors CXCR4, CX3CR1, and integrin α4β7 in addition to the early activation marker CD69. Consistent with previous observations, post-menopausal status was associated with significant increases in the frequency of CCR5 expression on CD4^+^ T cells isolated from the EM (**Figure 3A**), CX (**Figure 3B**), and ECX (**Figure 3C**). Increased α4β7 expression frequency was also observed on CD4^+^ T cells of the EM (**Figure 3A**) and CX (**Figure 3B**), but not the ECX (**Figure 3C**) following menopause, while CCR5 + α4β7 co-expression was observed to be greater only in the EM (**Figure 3A**). In contrast, no changes in CD4^+^ T cell expression of CXCR4, CX3CR1, or CD69 were observed for any tissue (**Figures 3A–C**).

We next evaluated whether observed changes in surface marker expression (**Figure 3A**) correlated with increased infection susceptibility in the EM (**Figure 3D**) by comparing the BaL-GFP infection frequency of specific receptor expressing cells between pre- and post-menopausal women. After controlling for changes in surface marker expression frequency, the infection frequency of CCR5, α4β7, CCR5 + α4β7, CXCR4, and CD69 expressing CD4^+^ T cells remained greater in the EM of women following menopause (**Figure 3D**). HIV-1 infection frequency of CX3CR1^+^ CD4^+^ T cells likewise trended greater in the EM of post-menopausal women but did not reach statistical significance (**Figure 3D**). In contrast to the EM, no changes in infection frequency were observed with menopause in the CX (**Figure 3E**) nor ECX (**Figure 3F**) for any of the markers measured. Taken together this data indicates that despite broad increases in the expression of previously described determinates of CD4^+^ T cell HIV-1 infection susceptibility across all three tissue types following menopause, the increased expression of CCR5 and α4β7 is only associated with increases in EM CD4^+^ T cell infection susceptibility.

### Menopause alters T helper cell subset composition and density in the EM but does not appear to drive increased HIV-1 infection frequency

**Figure 4:**
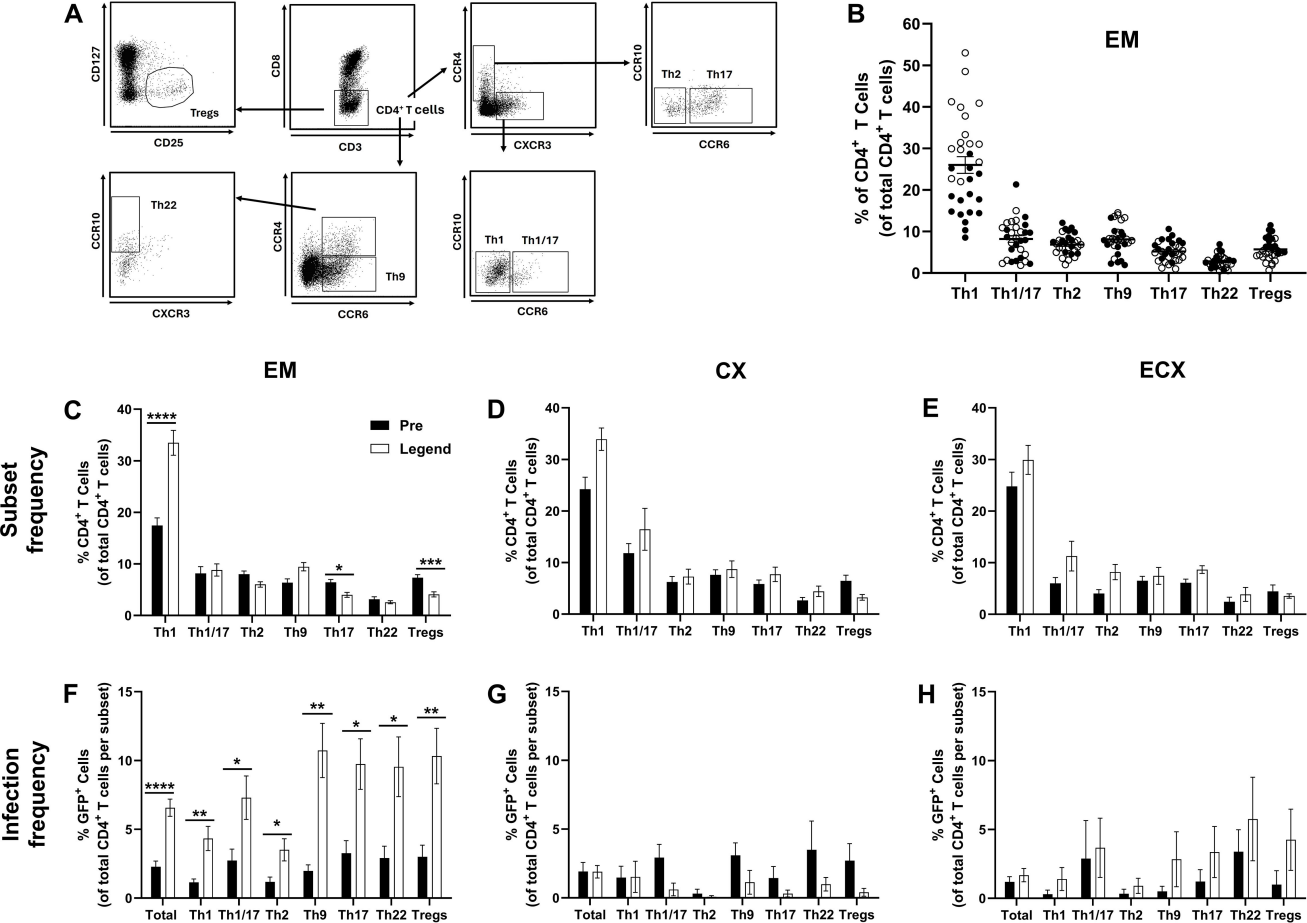
Effect of menopausal status on T helper cell subset composition and HIV-1 infection frequency in the EM. Gating strategy for the identification of Th1, Th1/17, Th2, Th9, Th17, Th22, and regulatory T cells [Tregs; (**A**)]. Relative T helper cell subset composition in the EM **(B)**. Menopausal associated changes in CD4^+^ T helper cell subset composition in the EM **(C)**, the CX **(D)**, and the ECX **(E)**. The effect of menopause on CD4^+^ T helper cell subset infection frequency in the EM **(F)**, CX **(G)**, and ECX **(H)**. Data from pre-menopausal patients are shown with solid black bars while data from post-menopausal patients are shown with open black bars. Data represent the mean ± SEM with significant differences determined by Mann-Whitney *U* test using the Holm-Šídák correction for multiple comparisons. *P < 0.05, **P < 0.01, ***P < 0.001, and ****P < 0.0001.

Functional polarization has been shown to further influence CD4^+^ T cell HIV infection susceptibility with CCR6 expressing Th17 and Th22 cells identified as preferential targets of HIV infection in the FRT (25, 26, 41). As prior work by our group suggested that the frequency of CCR6 expressing CD4^+^ T cells increases after menopause in the EM, but not the CX and ECX (25), we next sought to determine whether shifts in the relative composition of T helper cell subsets could be driving increased infection frequency in the EM. To test this hypothesis, the relative frequencies and densities of Th1 cells, Th1/17 cells, Th2 cells, Th9 cells, Th17 cells, Th22 cells, and regulatory T cells (Tregs) were quantified by surface maker expression as described previously (42, 43) using the gating strategy shown in (**Figure 4A**).

Th1 cells were the predominant CD4^+^ T helper cell subset in the mucosa of the EM (mean frequency of 26.0%), with reduced, but roughly equal, mean frequencies of Th1/17 cells (8.2%), Th2 cells (6.8%), Th9 cells (8.1%), Th17 cells (5.2%), and Tregs (5.7%; **Figure 4B**). Th22 cells were the least common T helper subset in the EM with a mean frequency of 2.8%. T helper subset compositions similar to that of the EM were observed in the CX (**Supplementary Figure 1A**) and ECX (**Supplementary Figure 1B**), with a greater mean frequency of Th1/17 cells observed in the CX (14.1%) relative to the EM (8.2%) and ECX (8.8%). Following stratification by menopausal status, the mean frequency of Th1 cells was observed to be greater following menopause in the EM (17.5% in pre-menopausal patients versus 33.5% in post-menopausal patients; **Figure 4C**), which was offset by decreases in the mean frequency of Th17 (6.4% vs 4.0%) and Tregs (7.3% vs 4.1%). No changes in Th1/17, Th2, Th9, or Th22 cell frequency were observed following menopause in the EM. Unlike the EM, no changes in the frequency of any phenotype was observed in the CX (**Figure 4D**) or ECX (**Figure 4E**) though a non-significant (p = 0.09) increase in Th1 cell frequency was observed in the CX following menopause.

To further evaluate the effects of menopause on T helper subset composition in the female reproductive tract, we next compared the mean densities of Th1 cells, Th1/17 cells, Th2 cells, Th9 cells, Th17 cells, Th22 cells, and Tregs in EM, CX, and ECX between pre- and post-menopausal women. Following stratification by menopausal status, mean densities of Th2 (2.44 x 10^4^ cells/g vs 0.67 x 10^4^ cells/g), Th17 (1.94 x 10^4^ cells/g vs 0.44 x 10^4^ cells/g), Th22 (0.67 x 10^4^ cells/g vs 0.29 x 10^4^ cells/g), and Tregs (1.79 x 10^4^ cells/g vs 0.47 x 10^4^ cells/g) were all greater in the EM (**Supplementary Figure 2A**) of pre- relative to post-menopausal women. In contrast, no changes in the mean density of any T helper subset were observed with menopausal status in the CX (**Supplementary Figure 2B**) and ECX (**Supplementary Figure 2C**).

To determine whether observed changes in T helper subset composition (**Figure 4C**) correlated with increased infection susceptibility in the EM (**Figure 4F**) we next compared the BaL-GFP infection frequency of specific T helps subsets between pre- and post-menopausal women. After controlling for changes in T helper cells subset composition, the mean frequency of infection remained significantly greater for Th1 (4.3% vs 1.2%), Th1/17 (8.1% vs 2.7%), Th2 (3.5% vs 1.2%), Th9 (10.7% vs 2.0%), Th17 (9.7% vs 3.3%), Th22 (9.5% vs 2.9%), and Tregs (10.3% vs 3.0%) in the EM of post-menopausal women relative to pre-menopausal women (**Figure 4F**). In contrast, no changes in infection frequency were observed for any specific T helper subset in the CX (**Figure 4G**) nor ECX (**Figure 4H**) with menopausal status. Thus, phenotypic changes in T helper subset composition are unlikely to explain increases in HIV-1 infection susceptibility in CD4^+^ T cells isolated from the EM.

### E_2_ treatment reduces endometrial CD4^+^ T cell infection frequency following menopause independent of changes in CCR5 expression

Menopause is associated with dramatic declines in the concentrations of both progesterone and estradiol (E_2_) (29). Because E_2_ is known to reduce CD4^+^ T cell HIV-1 infection susceptibility *in vitro* (40, 44), we next assessed whether E_2_ treatment could reverse the observed increases in endometrial CD4^+^ T cell infection susceptibility following menopause as described in the methods.

**Figure 5:**
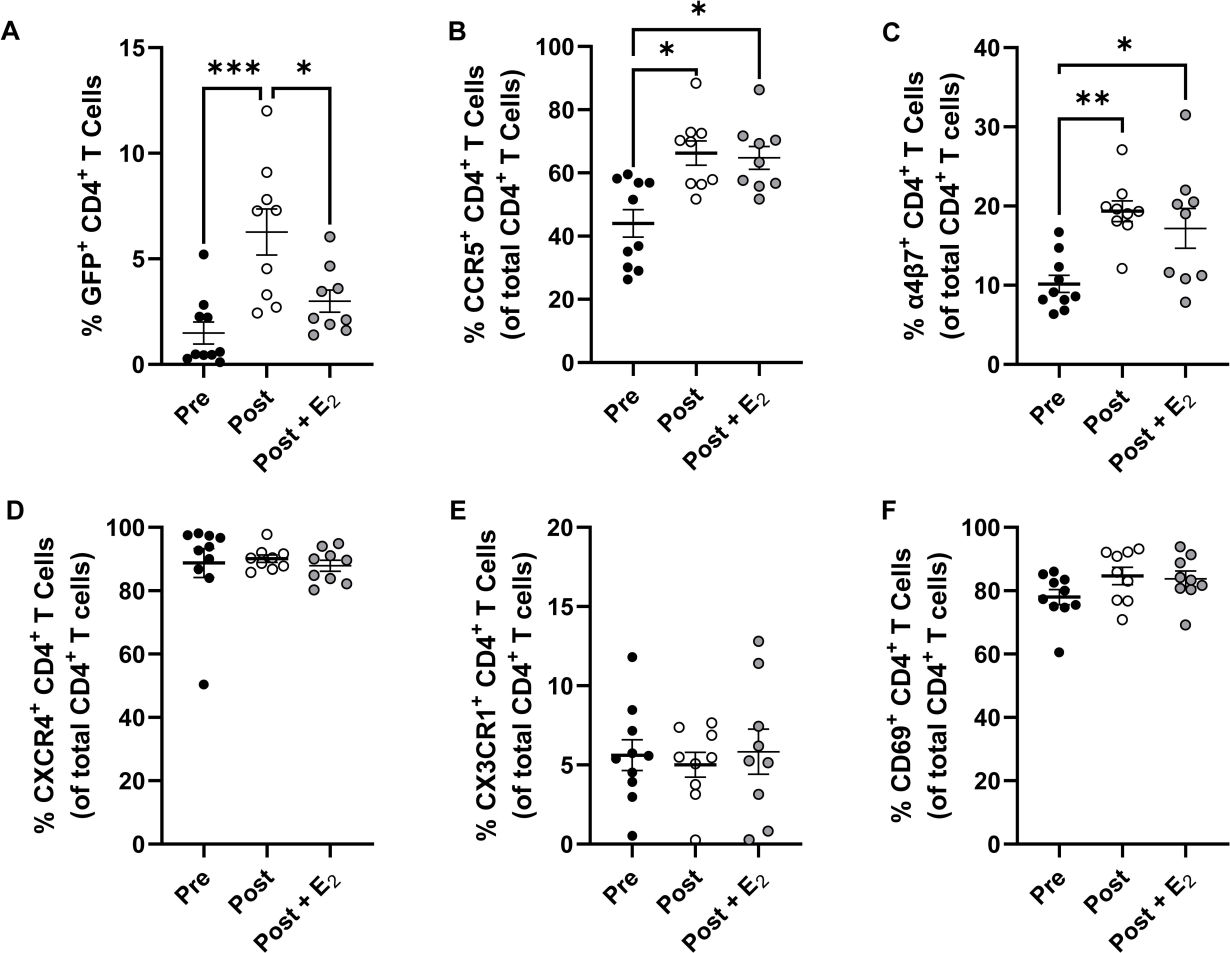
Effect of E_2_ treatment on endometrial CD4^+^ T cell HIV-1 infection susceptibility and select surface marker expression. Comparison of HIV-1 infection susceptibility of CD4^+^ T cells isolated from the EM of pre-menopausal and post-menopausal women infected in the presence or absence of E_2_ **(A)**. Comparison of CCR5 **(B)**, α4β7 **(C)**, CXCR4 **(D)**, CX3CR1 **(E)**, and CD69 **(F)** expression on CD4^+^ T cells isolated from the EM of pre-menopausal and post-menopausal women infected in the presence or absence of E_2_. Data from pre-menopausal patients are shown with back circles, data from post-menopausal patients are shown with open circles, and data from E_2_-treated post-menopausal samples are shown with gray filled circles. Data represent the mean ± SEM with significant differences determined by 2-way ANOVA followed by Tukey’s multiple comparison tests. *P < 0.05, **P < 0.01, and ***P < 0.001.

In the absence of E_2_ treatment, mean CD4^+^ T cell infection frequency was again significantly greater in the EM of post-menopausal versus pre-menopausal women (6.3% versus 1.7%; **Figure 5A**). In contrast, E_2_ treatment of post-menopausal cells significantly reduced the mean infection frequency by 52.4% (from 6.3% to 3.0%) in CD4^+^ T cells isolated from post-menopausal patients. Unlike with the EM, no significant differences were observed with either menopause or E_2_ treatment in CD4^+^ T cell infection frequency in the CX (**Supplementary Figure 3A**) or ECX (**Supplementary Figure 3B**).

To evaluate how E_2_ could be reducing EM CD4^+^ T cell HIV-1-infection frequency following menopause, the expression of CCR5, integrin α4β7, CXCR4, CX3CR1, and CD69 on these cells were measured by multicolor flow cytometry. While the expression frequencies of both CCR5 (**Figure 5B**) and α4β7 (**Figure 5C**) were greater on endometrial CD4^+^ T cells following menopause, unexpectedly, we found that their expression was not altered with E_2_ treatment. As before, the expression of CXCR4 (**Figure 5D**), CX3CR1 (**Figure 5E**), and CD69 (**Figure 5F**) did not differ between pre- and post-menopausal women and their expression frequencies in post-menopausal women were likewise not altered following E_2_ treatment. Taken together, E_2_ treatment can reduce menopausal associated increases in EM CD4^+^ T cell HIV-1 infection susceptibility *in vitro*. This reduction in infection frequency was independent of changes in the expression of CCR5, α4β7, and other previously described surface determinates of HIV-1 infection susceptibility.

## Discussion

Little is known of how menopause alters female susceptibility to infection with HIV and other sexually transmitted infections. In the present study, we demonstrate that while the frequency of HIV target cells in the EM decreases following menopause, their susceptibility to HIV-1 infection concurrently increases. While this was accompanied by changes in several previously described determinants of CD4^+^ T cell HIV infection susceptibility, notably increases in the expression frequency of the HIV coreceptors CCR5 and integrin-α4β7, neither appears to directly correlate with observed increases in infection frequency. Likewise, shifts in T helper cell subset composition and density were observed in the EM, but not the CX nor ECX, but were similarly not associated with increased HIV-1 infection frequency. Moreover, these studies further indicate that treatment with E_2_, which suppresses endometrial CD4^+^ T cell infection by HIV-1, does so without affecting co-receptor expression. Taken together, these studies demonstrate an unexpected complexity in post-menopausal increases in HIV-1 infection susceptibility. Lastly, these studies demonstrate that postmenopausal changes resulting in increased HIV-1 susceptibility are unique to the EM and are distinct from those changes seen in the CX and ECX. These findings provide valuable insight into how menopause alters the immune system of the FRT and suggests that post-menopausal women may be at an increased risk of HIV-1 infection.

Observed increases in CD4^+^ T cell CCR5 expression in the reproductive tract of post-menopausal women have previously been proposed to contribute to elevated HIV infection risk in this population (25, 35). Although we likewise observed broad increases in CCR5 expression frequency across all three tissue types following menopause, elevated CCR5 expression was only associated with increased CD4^+^ T cell infection frequency in the EM. In contrast, elevated CCR5 expression was not associated with increased CD4^+^ T cell infection frequency in the CX and ECX. This discordance across the tissues suggests that increased CD4^+^ T cells infection frequency in the EM is not explained by observed increases in CCR5 expression following menopause. Further, CCR5 expressing CD4^+^ T cells isolated from the EM of post-menopausal women remained more susceptible to HIV-1 infection relative to those of pre-menopausal patients. Though indirect, taken together these findings strongly suggest that observed increases in infection frequency in post-menopause women are site specific and not the result of changes in CCR5 expression frequency. Whether CCR5 expression frequency changes following menopause in a CD4^+^ T cell subset-specific manner requires further elucidation.

Significant increases in CD4^+^ T cell integrin α4β7 expression frequency was also observed in the EM and CX. Interestingly, these changes paralleled increased infection frequency in the EM but not the CX. To the best of our knowledge, we are the first to observe changes in α4β7 expression on CD4^+^ T cells of the EM following menopause. Prior work suggests that integrin α4β7 expression increases HIV-1 infection susceptibility of cervical, gut derived, and peripheral blood CD4^+^ T cells (20, 24, 45–47). Consistent with this, in the current study, α4β7 expressing CD4^+^ T cells were observed to be preferential targets of HIV-1 infection across all three FRT tissue types (**Figure 3**), particularly when co-expressed with CCR5. However, α4β7 expressing CD4^+^ T cells isolated from the EM of post-menopausal remained significantly more susceptible to HIV-1 infection relative to those of pre-menopausal patients, suggesting that increased HIV-1 infection susceptibility was instead driven by other unknown factors. In combination, these observations provide indirect evidence that increased α4β7 expression frequency is not driving increased EM CD4^+^ T cell HIV-1 infection susceptibility following menopause. As increased CD4^+^ T cell α4β7 expression has been associated with accelerated intra-host HIV-1 dissemination and AIDS progression in pre-menopausal women (46), further studies are needed to determine whether increased α4β7 expression likewise accelerates disease progression in women infected following menopause.

In the present study we observed greater CD4^+^ T cell infection frequencies in the EM relative to the CX and ECX, which was primarily driven by increased susceptibility in tissue samples isolated from post-menopausal women. Previous work by our group has suggested that CD4^+^ T cell HIV-1 infection susceptibility may be greater in the ECX relative to the EM (25). This discrepancy is likely due to limited sample sizes of the prior study in addition to differences in both experimental design and patient population characteristics. While both studies used variants of HIV-1 BaL, our previous study infected CD4^+^ T cells with a 10-fold lower viral concentration (MOI of 0.1 versus 1.0) and quantified the percentage of infected cells by intracellular p24 staining rather than by GFP expression. These differences highlight the potential for both the HIV variant involved and the size of the viral inoculum to influence preferences for infecting specific subsets of target cells. The potential for viral inoculum characteristics, including the viral strain evaluated (e.g., transmitted/founder viruses), to influence menopause-associated changes in HIV infection outcomes cannot be excluded and requires further study.

Consistent with other recently published work by our group (48), we observed notable shifts in T helper cell subset composition and density exclusively in the EM, including increases in Th1 cell frequency offset by decreases in the frequencies of both Th17 and Treg cells. Unexpectedly, in the current study we found that these changes do not appear to be associated with direct changes in CD4^+^ T cell infection susceptibility, with CD4^+^ T cells of post-menopausal women remaining more susceptible to HIV-1 infection regardless of T helper subset assessed. Another possibility is that reductions in EM Treg frequency and density alters the endometrial inflammatory environment more broadly, thus non-specifically increasing cellular susceptibility to HIV-1 infection. Prior work by others suggests that greater Treg frequency in the peripheral bloodstream is associated with reduced CD4^+^ T cell HIV-1 infection susceptibility (19), while increased cervical Treg frequency is associated with both decreased genital inflammation and reduced HIV-1 infection target cell abundance (49). Consistent with this, enhanced *ex-vivo* HIV-1 replication in ectocervical tissues of post-menopausal women has been previously correlated with increased concentrations of pro-inflammatory mediators (32). As reductions in CD4^+^ T cell expression of CTLA-4 have similarly been observed in the EM, but not the CX and ECX (48), further work will be needed to determine if and how observed reductions in Treg numbers following menopause alter the inflammatory environment of the EM. Whether HIV coreceptor and activation marker expression changes following menopause on Tregs was not evaluated in the current study but warrants future study.

Menopause is associated with dramatic declines in the concentrations of estrogens in the FRT (29) and prior work by our group and others has demonstrated that exogenous estrogen treatment can reduce HIV infection susceptibility in a variety of *in vivo* and *ex vivo* models; particularly in the context of hypoestrogenic states (40, 44, 50–52). Consistent with this, in the current study, treatment of mixed cell suspensions with high, but physiologically relevant concentrations of exogenous E_2_ significantly reduced the EM CD4^+^ T cell infection frequency in samples from post-menopausal women independent of changes in CCR5 and α4β7 integrin expression. While estrogen mediated reductions in cellular HIV infection susceptibility have been previously demonstrated by us and others to be independent of changes CCR5 expression (40, 50), our findings now extend this by suggesting that the effects of E_2_ treatment on CD4^+^ T cell HIV-1 infection susceptibility is likewise independent of changes in α4β7 integrin expression. Moreover, our findings are consistent with previously reported receptor expression independent mechanisms including competitive CCR5 ligand secretion (40), inhibition of post-entry intracellular viral trafficking (44), and activation of antiviral pathways (50). Whether increased endometrial CD4^+^ T cell infection frequency following menopause is driven by declines in estrogen concentration, or aging more broadly, remains unclear. These findings contribute to the body of literature suggesting that exogenous estrogen treatment may reduce HIV infection risk in post-menopausal women, but further studies will be needed to elucidate the mechanism by which this occurs.

Unexpectedly, we uncovered a tissue-specific effect of menopausal status on HIV-1 infection susceptibility, with changes in HIV-1 infection target cell density and CD4^+^ T cell infection frequency only observed in the EM and not the CX nor ECX. One explanation for this finding is that in reproductive age women, the EM plays a unique role in facilitating pregnancy with inflammatory immune responses tightly regulated by sex hormones allowing for reproductive success (30). With menopause, and the resulting declines in sex hormone concentrations, reductions in this tolerogenic environment are observed in conjunction with the loss of natural reproductive function (30, 53). Alternatively, a greater emphasis on protection against foreign pathogens is observed in the CX and ECX which varies relatively little with menopausal status (54). Given the role Tregs play in both HIV infection susceptibility and facilitating fetal tolerance in the EM (55), our findings suggest that EM-specific reductions in Treg frequency following menopause may be a possible driver of the tissue-specific effects of menopausal status on endometrial CD4^+^ T cell HIV-1 infection frequency.

While the precise mechanism(s) driving increased endometrial CD4^+^ T cell susceptibility to HIV-1 infection following menopause remain unclear, increases in direct cellular susceptibility to infection are likely to be just one of several changes resulting in increased HIV-1 infection susceptibility in the reproductive tract of post-menopausal women. Prior research by others suggest that cervicovaginal epithelial thickness is reduced following menopause which occurs in conjunction with decreasing vaginal levels of the epithelial tight junction protein E-cadherin and suggests that mucosal barrier function against HIV infection may be reduced (56). Likewise, TLR8 signaling induced neutrophil extracellular net formation has been observed to be reduced in response to HIV stimulation in the reproductive tract of post- relative to pre-menopausal women (57). Moreover, concentrations of antiviral and inflammatory mediators in the lower reproductive tract have been shown to broadly decrease following menopause (56, 58) which may increase (33, 56) or decrease (58) HIV infection susceptibility depending on the methods used and the characteristics of the population being studied. Our findings extend these observations by further demonstrating that the susceptibility EM CD4^+^ T cells to HIV-1 infection also increases following menopause. As interactions with dendritic cells (DCs) can promote CD4^+^ T cell infection (59), and the functions of endometrial DCs are altered following menopause (60), whether DCs and natural killer cells contribute to post-menopausal increases in HIV-1 infection susceptibility warrants future study. The degree to which the above changes are caused by menopause associated declines in hormone concentrations or increasing age more broadly also remains largely unknown. Taken together, these findings suggest that multiple biological changes likely drive epidemiologic observations of increased HIV-1 infection susceptibility following menopause.

In conclusion, our findings demonstrate that the susceptibility of EM CD4^+^ T cells to HIV-1 infection increases following menopause in conjunction with increases in several known determinants of cellular HIV infection susceptibility. Further studies are needed to fully elucidate whether and how these observed menopause-associated changes alter HIV-1 infection susceptibility. As the population continues to age, understanding how menopause, and female aging more broadly, alter HIV infection susceptibility will aid in the development of effective strategies to reduce HIV infections.

## Data Availability

The original contributions presented in the study are included in the article/**Supplementary Material**, further inquiries can be directed to the corresponding author.

## Supplementary material

Supplementary Figure 1 Tissue specific T helper cell subset composition in the CX and ECX. Relative T helper cell subset composition in the CX **(A)** and ECX **(B)**. Individual patient data are shown with squares for CX tissue and with triangles for ECX tissue. Solid black shapes indicate data from pre-menopausal patients and open shapes indicate data from for post-menopausal patients. Data represent the mean ± SEM.

Supplementary Figure 2 Effect of menopausal status on T helper cell subset density in the EM, CX, and ECX. Effects of menopause on the T helper cell subset density in the EM **(A)**, CX **(B)**, and ECX **(C)**. Solid shapes indicate samples from pre-menopausal patients while open shapes indicate samples from post-menopausal patients. Data represent the mean ± SEM with significant differences determined by Mann-Whitney *U* test using the Holm-Šídák correction for multiple comparisons. *P < 0.05, **P < 0.01, ***P < 0.001, and ****P < 0.0001.

Supplementary Figure 3 Effect of E_2_ treatment on CD4^+^ T cell HIV-1 infection susceptibility in the CX and ECX. Comparison of HIV-1 infection susceptibility of CD4^+^ T cells isolated from the CX **(A)** and ECX **(B)** of pre-menopausal and post-menopausal women infected in the presence or absence of E_2_. Individual patient data are shown with squares for CX tissue and with triangles for ECX tissue. Solid black shapes indicate data from pre-menopausal patients, open shapes indicate data from for post-menopausal patients treated with placebo, and data from E_2_-treated post-menopausal samples are shown with gray filled shapes. Data represent the mean ± SEM.
