# Supplementary figures and images for: Increases in the susceptibility of human endometrial CD4+ T cells to HIV-1 infection post-menopause are not dependent on greater viral receptor expression frequency
Source: Front Immunol. 2025 Jan 13;15:1506653. doi: 10.3389/fimmu.2024.1506653 (PMC11769835; doi:10.3389/fimmu.2024.1506653)

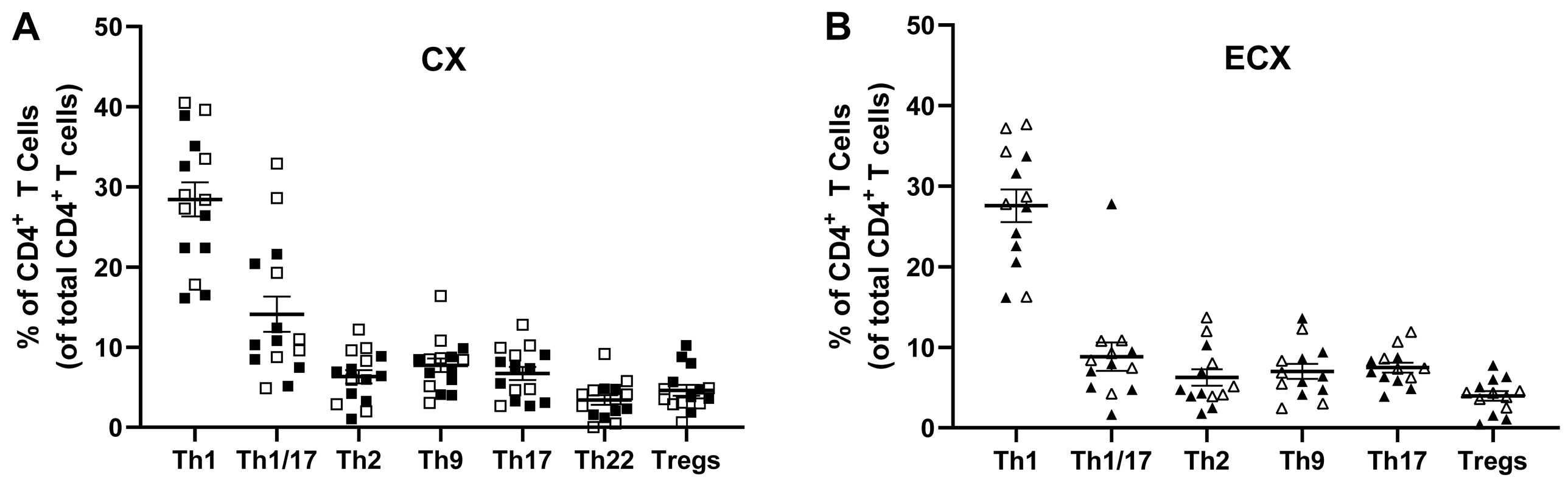

Supplement: Supplementary Figure 1 — Tissue specific T helper cell subset composition in the CX and ECX. Relative T helper cell subset composition in the CX (A) and ECX (B). Individual patient data are shown with squares for CX tissue and with triangles for ECX tissue. Solid black shapes indicate data from pre-menopausal patients and open shapes indicate data from for post-menopausal patients. Data represent the mean ± SEM. [file Image1.jpeg]

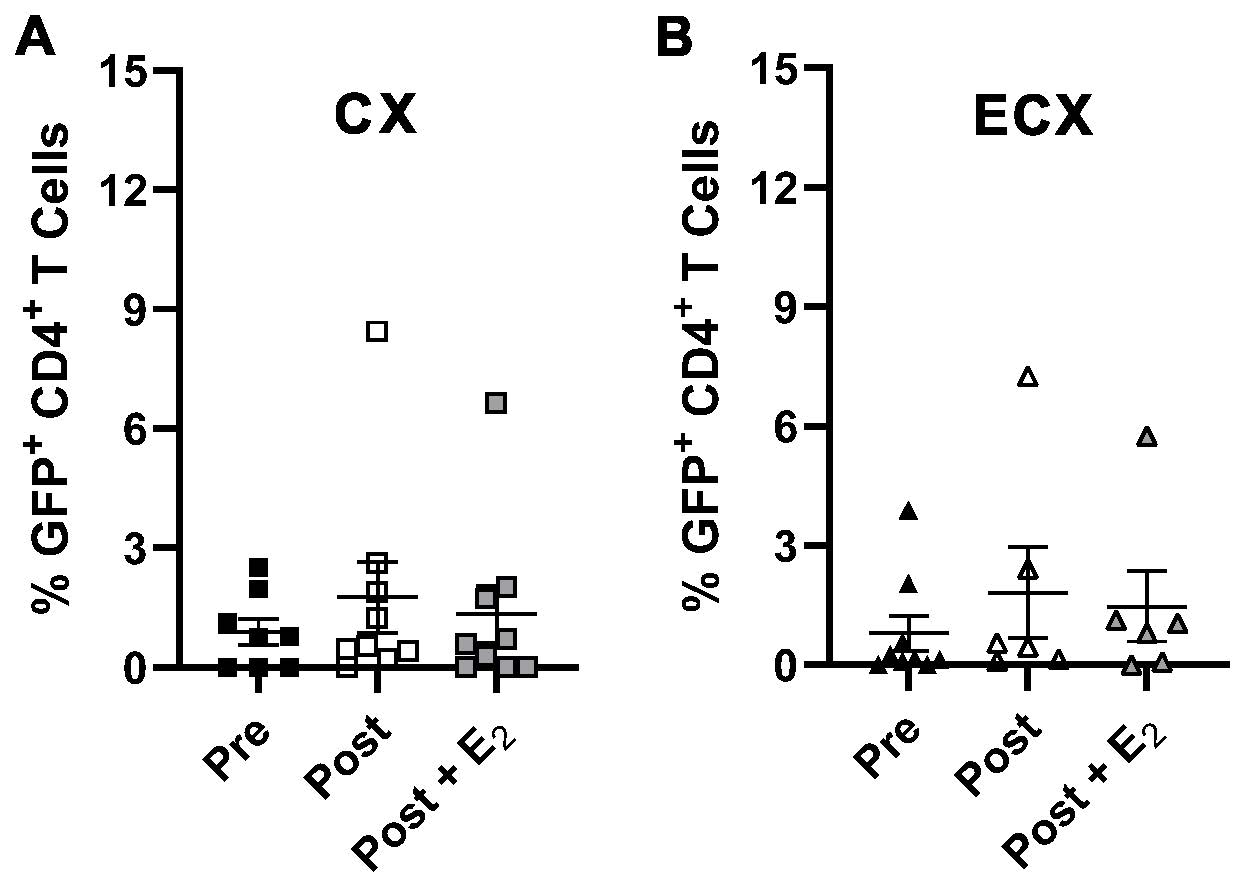

Supplement: Supplementary Figure 3 — Effect of E2 treatment on CD4+ T cell HIV-1 infection susceptibility in the CX and ECX. Comparison of HIV-1 infection susceptibility of CD4+ T cells isolated from the CX (A) and ECX (B) of pre-menopausal and post-menopausal women infected in the presence or absence of E2. Individual patient data are shown with squares for CX tissue and with triangles for ECX tissue. Solid black shapes indicate data from pre-menopausal patients, open shapes indicate data from for post-menopausal patients treated with placebo, and data from E2-treated post-menopausal samples are shown with gray filled shapes. Data represent the mean ± SEM. [file Image3.jpeg]
